# The Association Between Physical Activity and Peripheral Neuropathy in Diabetic Patients: A Cross-Sectional Multicenter Study From Saudi Arabia

**DOI:** 10.7759/cureus.34504

**Published:** 2023-02-01

**Authors:** Rawan Y AlKhotani, Sami A Al-Dubai, Mashael S Almeshaly, Abdulmajeed S Alautabi, Saleh F Maqulah, Zain J AlGhamdi, Zeyad S Alahmadi

**Affiliations:** 1 Neurosurgery Department, King Salman Bin Abdulaziz Medical City, Medina, SAU; 2 Joint Program of Preventive Medicine Post Graduate Studies, Ministry of Health, Medina, SAU; 3 Emergency Department, Alharam Hospital, Medina, SAU; 4 Family Medicine Department, Al Safiah Health Care Center, Medina, SAU; 5 Endocrinology Department, King Fahad Hospital, Medina, SAU

**Keywords:** saudi population, physical activity, nervous system, peripheral diabetic neuropathy, diabetes mellitus

## Abstract

Background

Peripheral diabetic neuropathy (PDN) is a serious consequence of diabetes mellitus (DM) that can impair quality of life and result in physical disability. This study aimed to investigate the relationship between physical activity and the severity of PDN among a sample of Saudi diabetic patients in Medina city, Saudi Arabia.

Methodology

A total of 204 diabetic patients participated in this multicenter, cross-sectional study. A validated self-administered questionnaire was distributed electronically to patients on-site during follow-up. Physical activity and diabetic neuropathy (DN) were assessed using the validated International Physical Activity Questionnaire (IPAQ) and the validated Diabetic Neuropathy Score (DNS), respectively.

Results

The mean (SD) age of the participants was 56.9 (14.8) years. The majority of the participants reported low physical activity (65.7%). The prevalence of PDN was 37.2%. There was a significant correlation between the severity of DN and the duration of the disease (p = 0.047). Higher neuropathy score was noticed in those with hemoglobin A1C (HbA1c) level ≥7 compared to those with lower HBA1c (p = 0.045). Overweight and obese participants had higher scores compared to normal-weight participants (p = 0.041). The severity of neuropathy decreased significantly when the level of physical activity increased (p = 0.039).

Conclusions

There is a significant association between neuropathy and physical activity, body mass index, duration of diabetes mellitus, and HbA1c level.

## Introduction

Diabetic neuropathy (DN) is a common complication associated with diabetes mellitus (DM) due to high blood sugar and high levels of fat which can damage the peripheral nerves [1,2]. The most common type of DN is peripheral diabetic neuropathy (PDN) which affects 25-50% of patients with diabetes [2]. Additionally, it can result in side effects such as infections, foot blisters, and ulcers. DN is classified as acute and chronic neuropathy. The acute form is associated with severe weight loss and can cause severe pain with or without sensory loss. In the acute type of DN, there is no motor sign, reflex loss, or nerve degeneration. With adequate control, it resolves completely within six months [3]. Chronic DN causes inflammation and demyelination of the nerves. It is characterized by a stocking-and-glove distribution sensory loss in the feet and legs, followed by the hands and arms. Signs and symptoms of chronic DN include numbness, temperature changes, tingling or burning sensation, muscle weakness, excessive sensitivity to touch, and mild gait abnormalities [4]. These signs and symptoms worsen at night [4]. It has been established that insulin resistance plays an important role in the development of peripheral neuropathy along with metabolic syndrome [5-7]. Previous studies reported that physical activity improved diabetes in general and prevented complications, such as PDN, by enhancing insulin sensitivity and increasing glucose uptake by the cells [6-8]. In addition, physical activity ameliorates the structure and function of muscles and nerves by renewing small sensory nerve damage [9]. Physical activity can prevent DN through a variety of mechanisms, including maintaining healthy blood glucose levels, increasing nerve blood flow, promoting axonal renewing, boosting neurotransmitter concentrations, and Na/K-ATPase, which plays a crucial role in nerve conduction velocity [7]. A recent systematic review reported that physical activity provided symptomatic relief in PDN, and its effects depend on the severity of the disease [8]. Current clinical practice recommendations suggest moderate-intensity or 75 minutes of vigorous-intensity aerobic physical activity each week [9].

There is a lack of studies in Saudi Arabia regarding the association between physical activity and the severity of PDN. This study aimed to investigate the relationship between physical activity and the severity of PDN among a sample of Saudi diabetic patients in Medina city, Saudi Arabia.

## Materials and methods

Study setting and participants

A total of 204 diabetic patients participated in this multicenter, cross-sectional study. Diabetic patients were enrolled from healthcare centers, diabetes clinics at King Fahad Hospital, and the Diabetic Center in Medina city, Saudi Arabia, from November to December 2022. Patients were included if they were registered as confirmed diabetic patients and aged more than 18 years with type I or type II DM. The minimum sample size required for this study was 200 patients.

Data collection tool

A validated, self-administered questionnaire was distributed electronically to patients on-site during follow-up. The questionnaire included three main sections. The first section included (a) sociodemographic data, such as age, gender, marital status, weight and height, education level, and income; and (b) clinical data such as the type of diabetes, duration of the disease, level of hemoglobin Ac1 (HbAc1), and type of medications. The second domain assessed physical activity using the validated Arabic version of the International Physical Activity Questionnaire (IPAQ) [10]. According to this questionnaire, participants were classified into three categories, namely, low, moderate, and high physical activity [10]. The third section assessed peripheral neuropathy using the validated Diabetic Neuropathy Score (DNS) [11]. The total score ranges from zero to nine, where 0-2 indicates no neuropathy, 3-4 mild neuropathy, 5-6 moderate neuropathy, and 7-9 indicates severe neuropathy.

Ethical approval

The study was approved by the Institutional Review Board (IRB), General Directorate of Health Affairs in Medina (approval number: lR822-101, dated November 15, 2022). Informed written consent was obtained from all participants, and confidentiality was assured.

Statistical analysis

The statistical analysis was done using SPSS® version 22.0 (IBM Corp., Armonk, NY, USA). Descriptive statistics were used to obtain frequency and percentage for categorical variables and mean and SD for continuous variables. A test of normality was performed for the total DNS. Analysis of variance (ANOVA) test and t‑test were used to assess the associations between the neuropathy score and sociodemographic and clinical variables. P-values <0.05 indicated statistical significance.

## Results

A total of 204 patients participated in this study. The mean (SD) age of the participants was 56.9 (14.8) years, ranging from 18 to 86 years. The majority were women (54.4%), married (73.5%), and had a family income of less than 5,000 SAR (56.9%). Approximately, one-third of the participants were overweight (32.4%), and 37.7% of the participants were obese (Table 1). Most patients reported a low physical activity score (65.7%), while 27.5% and 6.9% reported moderate and high physical activity scores, respectively. Most participants had type 2 DM (88.2%), had a disease duration of more than 10 years (62.3%), and took metformin (70.6%). Regarding the type of medications, taking tablets was reported by 42.2%, followed by insulin (35.8%). The last HbA1c level was ≥7 in most patients (81.4%), and the last test was done in the previous three months by 70.6% of the patients. More than half (56.4%) of the participants took vitamin B12 (Table 2).

**Table 1:** Sociodemographic characteristics of the participants.

Variable	N	%
Age (years)		
18–30	19	9.3
31–50	35	17.2
>50	150	73.5
Gender		
Men	93	45.6
Women	111	54.4
Education		
Read/write or primary	92	45.1
Secondary/tertiary	52	25.5
Diploma	9	4.4
University	51	25.0
Marital status		
Married	150	73.5
Non-married	54	26.5
Family income (SAR)		
<5,000	116	56.9
5,000–10,000	35	17.2
>10,000	53	26.0
Body mass index (kg/m^2^)		
Normal	61	29.9
Overweight	66	32.4
Obese	77	37.7
Physical activity		
Low	134	65.7
Moderate	56	27.5
High	14	6.9

**Table 2:** Clinical characteristics of the participants.

Variable	N	%
Type of diabetes		
Type 2	180	88.2
Type 1	24	11.8
Duration of diabetes (years)		
≤5	44	21.6
6–10	33	16.2
>10	127	62.3
Type of medication		
Insulin	73	35.8
Tablets	86	42.2
Both	18	8.8
No medication	27	13.2
Do you take metformin for diabetes?		
No	60	29.4
Yes	144	70.6
Result of the last hemoglobin A1c test		
<7	38	18.6
≥7	166	81.4
The last test for hemoglobin A1c		
≤3 months	144	70.6
>3 months	60	29.4
Do you take vitamin B12?		
No	89	43.6
Yes	115	56.4

DN was assessed by five items. More than half (59.3%) of the participants reported feeling burning or numbness, pain, cramps, or aches in the foot, or all of them. Approximately, one-third reported that the symptoms awakened them at night (33.8%). Symptoms worsened in the evening in 23.5% and in the day in 26.0% of the participants (Table 3). Regarding the prevalence of neuropathy, 43.6% had no neuropathy, 19.1% had mild neuropathy, 24.0% had moderate neuropathy, and 13.2% had severe neuropathy. Accordingly, 37.2% of the participants had PDN.

**Table 3:** Diabetic Neuropathy Score.

Variable	N	%
Do you feel burning or numbness, pain, cramps, aches in the foot, or all of them?		
No	83	40.7
Yes	121	59.3
Have the symptoms ever awakened you at night?		
No	135	66.2
Yes	69	33.8
What is the location of the symptoms?		
Calf	144	70.6
Foot	22	10.8
Both	38	18.6
What is the timing of the symptoms?		
No pain	103	50.5
In the evening	48	23.5
During the say	53	26.0
How are the symptoms relieved?		
Not relieved	10	4.9
On sitting or lying down	49	24.0
On walking	42	20.6
On standing	8	3.9
No pain	95	46.6
Neuropathy		
No	89	43.6
Mild	39	19.1
Moderate	49	24.0
Severe	27	13.2

Regarding factors associated with neuropathy, the mean neuropathy score increased significantly as the duration of the disease increased (p = 0.047). Patients with an HbA1c level ≥7 had higher neuropathy scores compared to those with lower HbA1c (p = 0.045). Overweight and obese participants had higher scores compared to normal-weight participants (p = 0.041) (Table 4). The severity of neuropathy decreased significantly when the level of physical activity increased (p = 0.039).

**Table 4:** Relationship between neuropathy and study variables.

Variable	Mean	Standard deviation (SD)	P-value
Age (years)			
18–30	3.2	2.27	0.302
31–50	3.3	2.27	
>50	3.7	2.33	
Gender			
Men	3.4	2.33	0.215
Women	3.8	2.29	
Education			
Read/write or primary	3.5	2.28	0.778
Secondary/tertiary	3.9	2.50	
Diploma	3.4	2.07	
University	3.6	2.26	
Marital status			
Married	3.5	2.20	0.316
Non-married	3.9	2.60	
Family income (SAR)			
<5,000	3.8	2.28	0.534
5,000–10,000	3.4	2.30	
>10,000	3.4	2.40	
Body mass index (kg/m^2^)			
Normal	3.4	2.41	0.041
Overweight	3.6	2.31	
Obese	3.8	2.26	
Physical activity			
Low	3.9	2.26	0.039
Moderate	3.6	2.42	
High	3.1	2.32	
Type of diabetes			
Type 2	3.5	2.25	0.129
Type 1	4.3	2.69	
Duration of diabetes (years)			
≤5	2.9	2.02	0.047
6–10	3.5	2.55	
>10	3.9	2.32	
Result of the last hemoglobin A1c			
<7	3.3	2.19	0.045
≥7	3.9	2.34	
The last test for hemoglobin A1c			
≤3 months	3.7	2.27	0.231
>3 months	3.3	2.41	
Type of medication			
Insulin	3.8	2.32	0.474
Tablets	3.4	2.24	
Both	3.5	2.50	
No medication	4.1	2.42	
Do you take metformin for diabetes?			
No	3.8	2.42	0.391
Yes	3.5	2.27	
Do you take vitamin B12?			
No	3.7	2.27	0.533
Yes	3.4	2.33	

## Discussion

This study aimed to investigate the relationship between physical activity and the severity of PDN among a sample of Saudi diabetic patients in Medina city. In this study, the prevalence of DN was 37.2%, which is comparable to the prevalence in the population of Gulf states (37.1%), but higher than that estimated worldwide (8.1-12.2%) [1,3,12].

This study found a significant association between DNS and physical activity, in which the severity of DN decreases as physical activity increases. A recent meta-analysis study that included 29 clinical trials reported that exercise is useful for patients with PDN [7].

Muscle contraction promotes blood glucose intake during exercise to enhance intramuscular glycogenolysis. Resting muscle absorbs glucose postprandially, depending on the level in the blood, to replace glycogen stores. Both routes enhance glucose uptake by the muscle after exercise [13]. Exercise has also been shown to enhance blood glucose management, decrease cardiovascular risk, and help in weight reduction [13]. For individuals with diabetes, current clinical practice guidelines recommend at least 150 minutes of weekly physical activity.

This study did not find an association between PDN and metformin use. There is conflicting evidence about the link between metformin-induced vitamin B12 deficiency and PDN. Some previous studies found that metformin was linked to a considerable decrease in vitamin B12 levels [14,15], whereas a recent study from Qatar found no difference in vitamin B12 levels between metformin and non-metformin users, and there was no difference in the prevalence of PDN or painful DN in type 2 DM patients with and without vitamin B12 deficiency [16]. In addition, a recent meta-analysis showed that only 10 out of 17 studies showed that metformin use led to vitamin B12 deficiency [17].

This study found no difference in neuropathy scores between those who take and those who did not take vitamin B12. This finding is consistent with a recent study from Turkey that found no difference in vitamin B12 levels in those with and without neuropathy [18]. Indeed, a study reported a lower prevalence of PDN in type 2 DM patients on metformin compared to those not on metformin [19]. However, a recent randomized clinical trial study found that treatment of patients with DN with 1 mg of oral vitamin B12 for 12 months improved the neurophysiological functions, pain score, and quality of life [20].

This study found a significant association between body mass index (BMI) and the severity of DN, in which the severity of neuropathy increased when BMI increased. Previous literature found that a BMI value of ≥25.0 kg/m^2^ in type 2 diabetic patients had a strong association with the development of peripheral neuropathy [4].

Limitations

Our study has some limitations. This study was conducted in one region which may limit its generalizability to the entire Saudi population. The cross-sectional design of the study may limit the causal relationship between the variables in the study.

## Conclusions

Most participants reported low physical activity (65.7%), and the prevalence of DN in this study was 56.3%. There is a significant association between neuropathy and physical activity, BMI, duration of DM, and HbA1c levels. It is recommended to raise the awareness of diabetic patients about the benefits of exercise in the prevention and management of neuropathy.
